# Modeling Parkinson’s Disease Neuropathology and Symptoms by Intranigral Inoculation of Preformed Human α-Synuclein Oligomers

**DOI:** 10.3390/ijms21228535

**Published:** 2020-11-12

**Authors:** Laura Boi, Augusta Pisanu, Maria Francesca Palmas, Giuliana Fusco, Ezio Carboni, Maria Antonietta Casu, Valentina Satta, Maria Scherma, Elzbieta Janda, Ignazia Mocci, Giovanna Mulas, Anna Ena, Saturnino Spiga, Paola Fadda, Alfonso De Simone, Anna R. Carta

**Affiliations:** 1 Department of Biomedical Sciences, University of Cagliari, 09042 Cagliari, Italy; boilaura92@hotmail.com (L.B.); mariaf.palmas@unica.it (M.F.P.); ecarboni@unica.it (E.C.); sattavalentina83@gmail.com (V.S.); mscherma@unica.it (M.S.); annaena96@gmail.com (A.E.); pfadda@unica.it (P.F.); 2 CNR Institute of Neuroscience, 09042 Cagliari, Italy; augusta.pisanu@in.cnr.it; 3 Centre for Misfolding Diseases, Department of Chemistry, University of Cambridge, CB2 1EW Cambridge, UK; gf203@hermes.cam.ac.uk; 4 CNR Institute of Translational Pharmacology, 09010 Cagliari, Italy; mariaantonietta.casu@ift.cnr.it (M.A.C.); ignazia.mocci@ift.cnr.it (I.M.); 5 Department of Health Sciences, Magna Graecia University, 88100 Catanzaro, Italy; janda@unicz.it; 6 Department of Life and Environmental Sciences, University of Cagliari, 09126 Cagliari, Italy; giovannamulas2@gmail.com (G.M.); sspiga@unica.it (S.S.); 7 Italian Neuroscience Institute (INN), 10126 Torino, Italy; 8 Department of Life Sciences, Imperial College London, London SW7 2AZ, UK; 9 Department of Pharmacy, University of Naples “Federico II”, 80131 Naples, Italy

**Keywords:** Parkinson disease, α-synuclein oligomers, neurodegeneration, neuroinflammation, microglia, motor deficits, cognitive impairment

## Abstract

The accumulation of aggregated α-synuclein (αSyn) is a hallmark of Parkinson’s disease (PD). Current evidence indicates that small soluble αSyn oligomers (αSynOs) are the most toxic species among the forms of αSyn aggregates, and that size and topological structural properties are crucial factors for αSynOs-mediated toxicity, involving the interaction with either neurons or glial cells. We previously characterized a human αSynO (H-αSynO) with specific structural properties promoting toxicity against neuronal membranes. Here, we tested the neurotoxic potential of these H-αSynOs in vivo, in relation to the neuropathological and symptomatic features of PD. The H-αSynOs were unilaterally infused into the rat substantia nigra pars compacta (SNpc). Phosphorylated αSyn (p129-αSyn), reactive microglia, and cytokine levels were measured at progressive time points. Additionally, a phagocytosis assay in vitro was performed after microglia pre-exposure to αsynOs. Dopaminergic loss, motor, and cognitive performances were assessed. H-αSynOs triggered p129-αSyn deposition in SNpc neurons and microglia and spread to the striatum. Early and persistent neuroinflammatory responses were induced in the SNpc. In vitro, H-αSynOs inhibited the phagocytic function of microglia. H-αsynOs-infused rats displayed early mitochondrial loss and abnormalities in SNpc neurons, followed by a gradual nigrostriatal dopaminergic loss, associated with motor and cognitive impairment. The intracerebral inoculation of structurally characterized H-αSynOs provides a model of progressive PD neuropathology in rats, which will be helpful for testing neuroprotective therapies.

## 1. Introduction

Parkinson’s disease (PD) belongs to the family of synucleinopathies, whose hallmark is the accumulation of misfolded aggregates of the protein alpha-synuclein (αSyn) in neuronal and non-neuronal brain cells [1]. The heterogeneous process of αSyn aggregation, which in vivo is associated with significant levels of phosphorylation of this protein, generates a variety of intermediate aggregated structures in vitro showing variable toxic potential [2,3,4]. Current evidence obtained by in vitro studies suggests that the size of αSyn species is a pivotal factor, and that short soluble oligomers (αSynOs) are the most toxic species against neurons, while the aggregation into larger assemblies is generally associated with reduced toxicity [4,5,6,7,8,9,10,11]. Accordingly, the αSyn pathology induced by αSyn fibrils in vivo, e.g., the extent of phosphorylated αSyn deposits (p129-αSyn) was inversely related to fibrils size [12]. Moreover, while aggregated αSyn activates microglia as a main component of PD neuropathology [13], oligomeric species seem to display the highest inflammatory properties in vitro and in vivo [11,13,14,15].

Recent studies have further investigated the structure–toxicity relationship in vitro of short human αSynOs (H-αSynOs) [7,16]. These studies have demonstrated that the binding modes with biological membranes are essential for initiating the mechanism of membrane disruption that underlies the toxicity of H-αSynOs against neurons [16]. In particular two essential elements of H-αSynOs were found in this mechanism, an N-terminal region acting as the membrane anchor [16,17,18,19,20,21,22,23,24], and a fibrillar core that effectively inserts into the hydrophobic layer of the membrane, and disrupts its integrity. These findings indicate that, in addition to size, topological properties such as secondary structure and tertiary structural elements are pivotal for gains in cellular toxicity by H-αsynOs [3,7,16,17,25,26]. Importantly, in a previous study the toxicity in vivo of H-αSynOs was demonstrated in the C. elegans over-expressing αSyn, where the incubation with antibodies specifically targeting the N-terminal region of the protein reduced the toxicity of the aggregated species [24].

Here, we assessed for the first time the neurotoxic action in vivo in rats of such purified toxic H-αsynOs, by investigating whether unilateral inoculation into the rat substantia nigra pars compacta (SNpc) would recapitulate, in a single model, the crucial hallmarks of PD, including cell loss, pathological deposits of p129-αSyn, and neuroinflammation, as well as motor impairment or non-motor symptoms.

Previous studies have investigated PD pathology after inoculation of preformed fibrils of αSyn (PFFs) into dopaminergic areas [12,27,28,29,30,31], reporting variable results in terms of neurodegeneration, motor impairment, and neuroinflammatory response [28,32]. Notably, the size distribution and structural properties of αSyn species were poorly characterized in most in vivo studies, or revealed an elevated degree of size-heterogeneity, leaving uncertain the toxic potential of the inoculated mix, and therefore cross-study replication and standardization. Importantly, the inoculation of PFFs of variable lengths into the rat substantia nigra pars compacta (SNpc) or striatum induced different amounts of intraneuronal deposits of p129-αSyn, and different degrees of nigrostriatal degeneration [12]. Here, we inoculated highly characterized H-αsynOs with unique features of size and structural homogeneity, as demonstrated by analytical ultracentrifugation (AUC), nuclear magnetic resonance (NMR), atomic force microscopy (AFM), and Fourier transform infrared (FT-IR) [7,16].

Since the progressive neurodegeneration in PD may be caused by pathological events that evolve crosswise disease stages, we here assessed, at progressive time-points, changes in H-αSynO-induced neuropathological hallmarks and associated symptoms. Specifically, we studied the formation of neuronal and microglial p129-αSyn in the injection area as well as in the striatum, as the main projection field of nigrostriatal dopamine neurons; we measured the nigral neuroinflammatory response as Iba-1, MHC-II, TNF-α, and IL-10 immunoreactivity (IR), in order to evaluate microglial activation and changes in cytokine profile induced by the infusion of H-αsynOs; in addition, to better characterize the microglial behavior in response to H-αsynOs, we performed a phagocytosis assay in vitro after pre-exposure of microglia cells to H-αsynOs. We investigated the toxic effect of H-αsynOs on dopaminergic neurons by measuring tyrosine hydroxylase (TH) IR and mitochondrial abnormalities and numbers in TH-positive cells in the SNpc, as well as by assessing striatal dopamine levels. Finally, we investigated whether the H-αsynOs infusion induced any motor or cognitive deficits.

## 2. Results

### 2.1. Assessment of H-αsynOs Optimal Volume and Concentration

**Figure 1:**
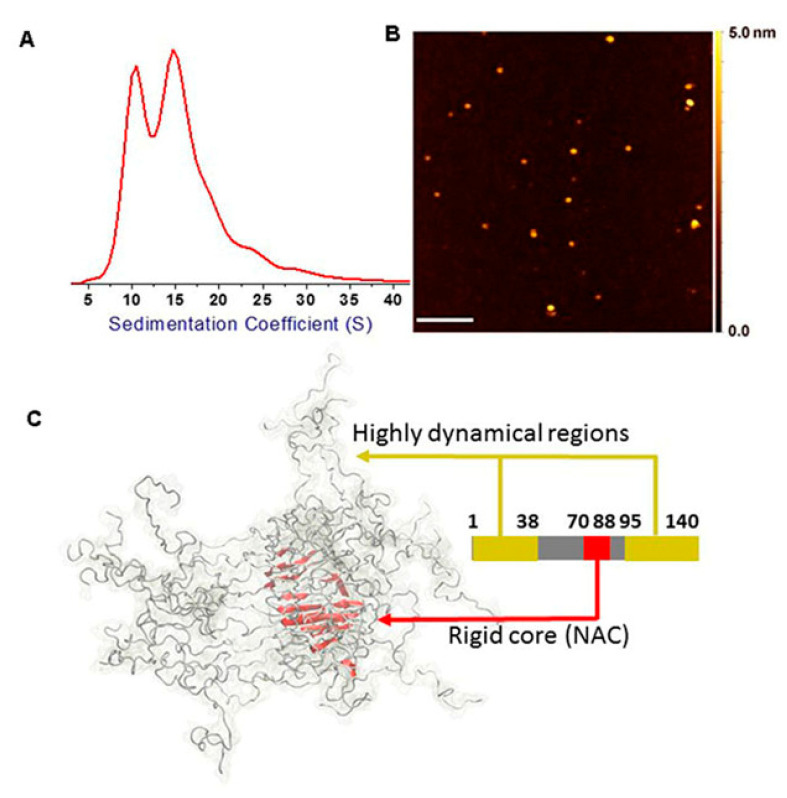
Structural properties of H-αSynOs employed in this work. The toxic oligomers employed in this work were previously characterized for their structural properties and designated as type-B* oligomers [16]. (**A**) Size distribution of the oligomers measured using analytical ultracentrifugation showed relatively high homogeneous properties. (**B**) Atomic force microscopy of type-B* oligomers deposited onto a mica surface. (**C**) Structural properties of type-B* oligomers analyzed using solid-state nuclear magnetic resonance (ssNMR). The oligomers showed a rigid fibrillar core in the region spanning residue 70 to 88 and two highly flexible regions exposed on the oligomer surface, namely the N-terminal C-terminal regions (residues 1 to 38 and 95 to 140). Membrane binding and disruption by these oligomers was found to be promoted by the interaction of the N-terminal region with the membrane surface in an amphipathic helical conformation, and by the insertion of the structured core into the interior of the membrane. All panels in this figure are adapted from [16].

**Figure 2:**
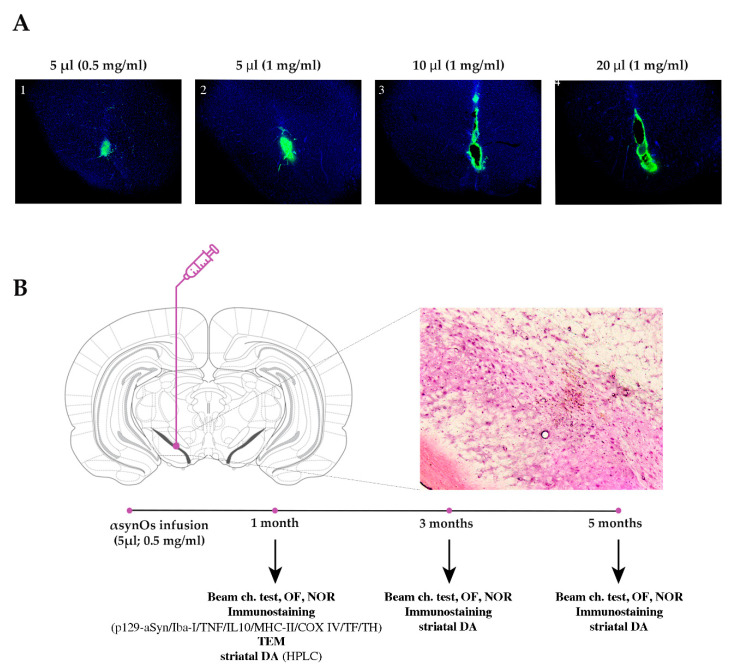
Representative pictures of the substantia nigra (SN) from rats infused with fluorescent H-αsynOs at increasing concentration and volume (**A**). Experimental design showing the site for H-αSynOs infusion in vivo, behavioral tests, and biochemical assays at progressive time-points (**B**). Insert in B shows hematoxylin and eosin (H&E) staining in a SN section adjacent to A-1. Beam ch. test: Beam challenging test; OF: open field; NOR: novel object recognition; TEM: transmission electron microscopy; TF: Thioflavin-S; DA: dopamine.

In this study we infused oligomers purified in vitro using H-αsynOs that induce significant toxicity when incubated with neuronal cultures [24]. These oligomers have a relatively high homogeneity in size, as shown by analytical ultracentrifugation (AUC), and atomic force microscopy (AFM) (Figure 1A,B), displaying an average length of 12 nm based on Cryo EM measurements [7,16]. Their homogeneity has allowed for a structural characterization using solid-state NMR, including the definition of highly dynamical and rigid-core regions of the assemblies [16]. Our previous work showed that these H-αsynOs have specific structural and topological properties at the membrane surface that promote a mechanism of disruption of the lipid bilayer, which is an upstream event generating cellular toxicity [16] (Figure 1C). In order to select the optimal H-αsynOs concentration and infusion volume in vivo that did not induce mechanical damage in the injection site, local deposits, or diffused staining (see methods), we performed a dose-finding experiment by infusing intranigrally fluorescent H-αsynOs at increasing concentrations and volume. The results indicated the concentration of 0.5 mg/mL (monomer equivalents) in 5 μL volume as the best combination, since it was not associated with any evident mechanical damage of the infused tissue, as confirmed by hematoxylin and eosin (H&E) staining, nor induced the deposition of local large aggregates, or diffused excessively in the infused area or along the injector trace (Figure 2A,B). In contrast, when H-αsynOs were infused at the concentration of 1 mg/mL in the same volume of 5 μL, large fluorescent deposits were observed at the infusion site, according with the notion that highly concentrated H-αsynOs rapidly aggregate and precipitate (Figure 2A). The concentration of 1 mg/mL infused in larger volumes of 10 and 20 μL, resulted in mechanical damage of the tissue, and fluorescence diffusion along the needle trace (Figure 2A).

### 2.2. Intranigral and Intrastriatal Deposits of Phosphorylated α-Synuclein

We evaluated whether the infusion of H-αsynOs into the SNpc induced the formation of pathological p129-αSyn within microglia and neurons in the infused SNpc, and whether the p129-αSyn pathology spread via dopaminergic fibers to the striatum; as this is the main SNpc projection area. p129-αSyn was analyzed as a frequently used marker for the pathological form of αSyn highly represented in protein aggregates.

#### 2.2.1. p129-αSyn in Microglia

**Figure 3:**
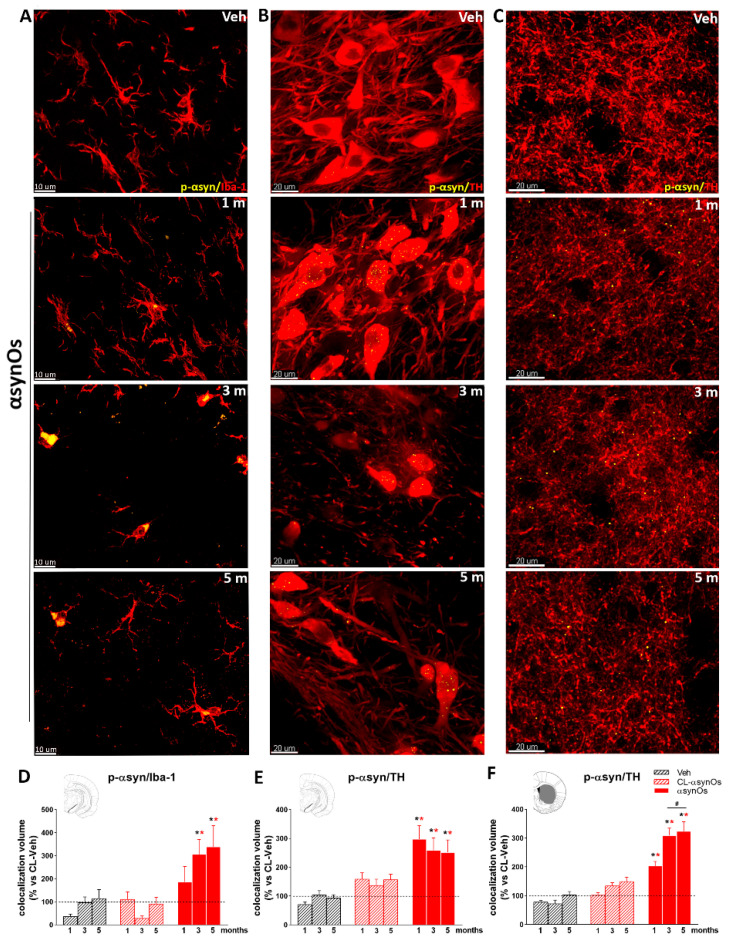
p129-αSyn aggregates in microglia and nigrostriatal neurons. Representative confocal images showing p129-αSyn (yellow) in Iba-1+ cells (red) (**A**), in TH+ cell bodies (red) (**B**) of the SNpc, and in TH+ fibers (red) of the striatum (**C**). Magnification 63X. Total volume occupied by p129-αSyn colocalized with Iba-1 (**D**) and TH (**E**) in the SNpc and colocalized with TH in the striatum at progressive time points post-infusion (**F**). Values are expressed as a percentage of the contralateral (CL) side of vehicle-injected animals (Veh) and represent the mean ± SEM. * *p* < 0.01 vs. Veh, * *p* < 0.001 vs. CL-H-αsynOs, # *p* < 0.0001 vs H-αsynOs, one month post infusion, by Tukey post-hoc test (*n* = 100–120 per group, 4–5 animals per group).

We measured p129-αSyn colocalized with Iba-1 positive (Iba-1+) cells and with TH positive (TH+) cells in the SNpc one, three, and five months after the H-αsynOs infusion. Qualitative and quantitative evaluation revealed the presence of few and small deposits of p129-αSyn inside Iba-1+ cells one month post infusion in the H-αsynOs-infused SNpc, and that significantly increased in number and became larger three and five months post infusion, as compared to all control groups (vehicle-injected SNpc and SNpc in the contralateral side, *p* < 0.01 and *p* < 0.001, respectively by Tukey post hoc) (Figure 3A,D; Supplementary Figure S1). p129-αSyn was almost absent in the control groups at all time points) (Figure 3A,D). Thioflavin S staining in Iba-1+ cells confirmed the presence of aggregates in microglia (Supplementary Figure S2).

#### 2.2.2. p129-αSyn in Dopaminergic Neurons

TH+ cell bodies into the SNpc displayed several small deposits of p129-αSyn as early as one month post infusion (* *p* < 0.01 vs. vehicle-injected animals (Veh), * *p* < 0.001 vs. the contralateral SNpc, by Tukey post hoc), suggesting that this was an earlier event preceding microglial uptake of p129-αSyn (Figure 3B,E). The colocalization volume of p129-αSyn remained stable after three and five months, with a slight, not significant decrease, likely related to the loss of dopaminergic neurons in the H-αsynOs-infused SNpc (Figure 3B,E; Supplementary Figure S1). Thioflavin S staining in TH+ cells confirmed the presence of aggregates in dopaminergic neurons of the SNpc (Supplementary Figure S2). When we measured the p129-αSyn colocalized with TH+ fibers within the striatum ipsilateral to the infused SNpc, we found an increase of small deposits of p129-αSyn one month post infusion (* *p* < 0.01 vs. Veh, * *p* < 0.001 vs. the contralateral SNpc, by Tukey post hoc) (Figure 3C,F), and that increased in number three- and five months post infusion (*p* < 0.0001 vs. one month post infusion (Figure 3C,F; Supplementary Figure S1). We did not find any p129-αSyn deposits in the TH+ cell bodies and fibers of the vehicle-injected control groups (Figure 3B,C,E,F), except for few deposits in the SNpc and striatum contralateral to H-αsynOs infusion, suggesting some spreading to the contralateral hemisphere (Figure 3E,F).

### 2.3. Neuroinflammation

We investigated neuroinflammatory responses in the SNpc as a neuropathological hallmark of PD. Since a persistent and dysregulated microgliosis has been reported in the brain of PD patients, we measured Iba-1 and major histocompatibility complex (MHC)-II IR as a marker of reactive microglia in the SNpc. Moreover, we measured the levels of cytokines TNF-α and IL-10 within Iba-1 positive cells, in order to investigate whether the microglia acquired a pro-inflammatory, or anti-inflammatory phenotype, respectively.

#### 2.3.1. Iba-1 and MHC-II IR

**Figure 4:**
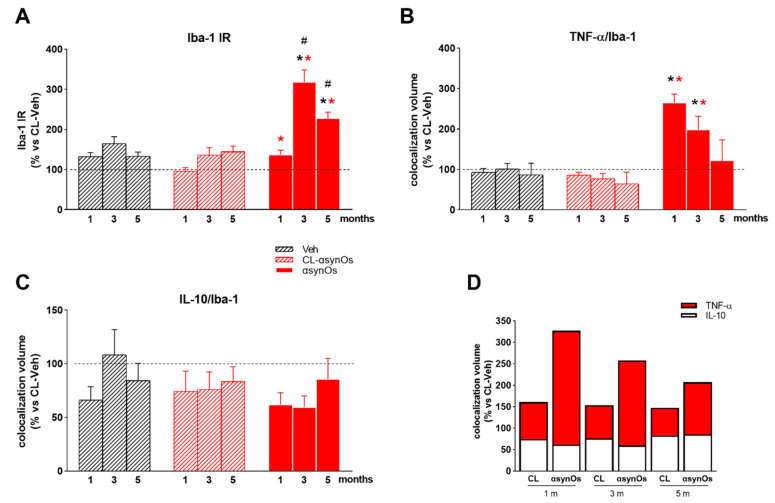
H-αSynOs induced reactive microgliosis and unbalanced cytokines expression in the SNpc. Total volumes occupied by Iba-1 (**A**), TNF-α, and IL-10 colocalized with Iba-1+ cells (**B,C**). Stacked bar chart shows the relative expression of TNF-α and IL-10 within Iba-1+ cells, which was unbalanced toward a proinflammatory phenotype following H-αsynO-infusion (**D**). Values are expressed as a percentage of the contralateral (CL) side of the vehicle-injected animals, and represent the mean ± SEM. * *p* < 0.01 vs. Veh, * *p* < 0.001 vs. CL-H-αSynOs, # *p* < 0.0001 vs. H-αSynOs, one month by Tukey post-hoc test (*n* = 100–120 per group, 4–5 animals per group).

**Figure 5:**
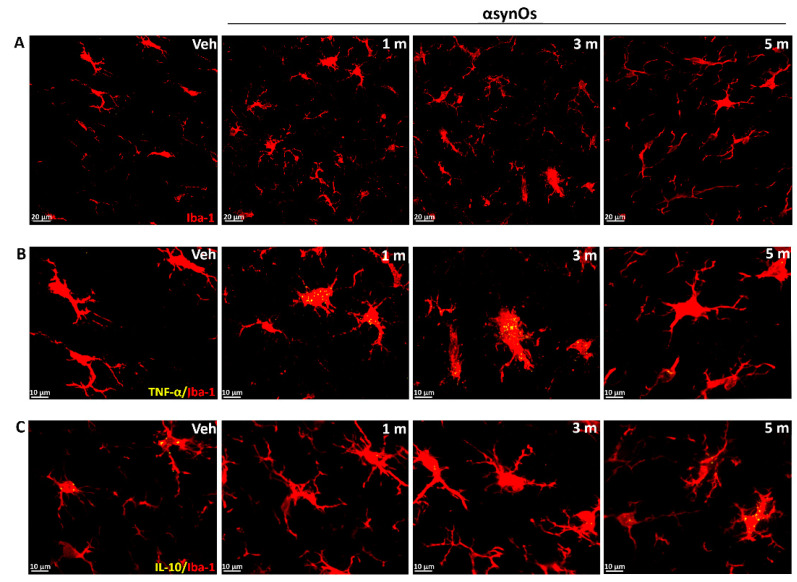
Representative images of Iba-1 (red, magnification 63×) (**A**), TNF-α (yellow) (**B**), and IL-10 (yellow) (**C**) colocalized with Iba-1+ cells (magnification 63×).

The H-αsynOs infusion induced an early and persistent microglial activation in the SNpc. Iba-1 IR was significantly increased compared to the contralateral side one month post infusion, and further increased three and five months post infusion (Figure 4A and Figure 5A) (see Figure 5 for statistics). Indicating an increase of inflammatory microglia, the number of cells stained for MHC-II was significantly increased in the SNpc infused with H-αsynOs, compared to the contralateral side (Supplementary Figure S3). Specifically, MHC-II staining was highly increased at one month post infusion compared to the contralateral SNpc, while it was still significantly higher, but to a much lesser extent, three- and five months post infusion. The vehicle-injected SNpc showed a slight increase of MHC-II staining at the earliest time-point only, likely related to the injection procedure (Supplementary Figure S3).

#### 2.3.2. Cytokines

The infusion of H-αsynOs into the SNpc induced a substantial increase of TNF-α levels within Iba-1-positive cells, compared to all control groups (SNpc in the contralateral side and vehicle-injected SNpc) (see Figure 5 for statistics). TNF-α was similarly increased one- and three months post infusion, while it returned to a value close to the control after five months (Figure 4B and Figure 5B). In contrast, levels of IL-10 within Iba-1+ cells were unchanged or slightly decreased ipsilaterally to H-αsynOs infusion after one, three, and five months (Figure 4C and Figure 5C). As a result, H-αsynOs infusion induced a disbalanced cytokine expression in the SNpc, as depicted in Figure 4D. While TNF-α and IL-10 displayed a 1:1 ratio in the SNpc, contralateral to H-αsynOs infusion, such a ratio was disrupted in the H-αsynOs-infused SNpc at all time-points analyzed, suggesting a shift of reactive microglia toward a pro-inflammatory phenotype.

### 2.4. Phagocytosis

**Figure 6:**
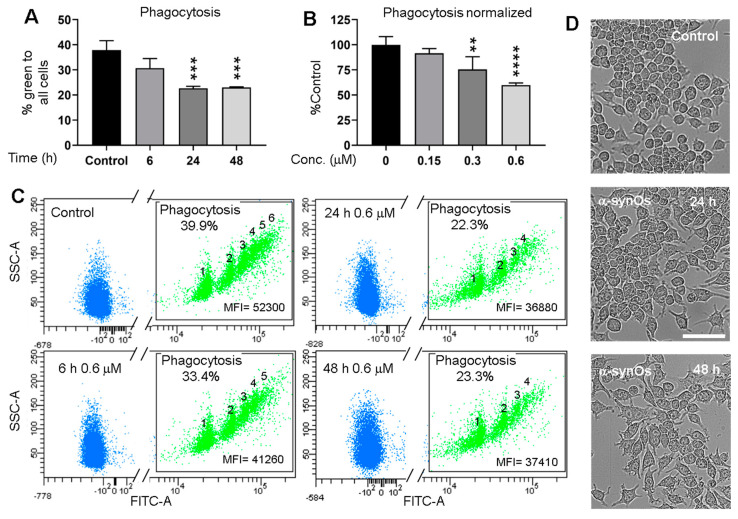
H-αSynOs impaired phagocytosis in MMGT microglia. (**A**) MMGT cells were exposed for different periods to 0.6 µM αsynOs or (**B**) to different doses of H-αSynOs for 24 h, and then subjected to phagocytosis assay with green fluorescent beads for 2.5 h. The percentage of green cells containing one or more phagocytosed beads was assessed by flow-cytometry and normalized (**B**), or not (**A**) to % phagocytosis in control cells. Graphs show the mean of 3 independent experiments +/- SD. **, ***, **** statistically significant differences at *p* < 0.05, 0.01, 0.0001 respectively, analyzed by Tukey post-hoc test. (**C**) Representative flow cytometry scatter plots showing green fluorescence (FITC) and side scatter (SSC) parameters of cells pre-treated for 6, 24, or 48 h with 0.6 µM H-H-αSynOs, and subjected to phagocytosis assay as in A. Green populations of cells containing 1, 2, 3, up to 6 beads per cell, % green cells with respect to total events and mean fluorescence intensity (MFI) were indicated for each scatter plot. (**D**) Microphotographs of MMGT cells treated as in C, before phagocytosis assay. Bar 100 µm. Note the different morphology of microglia in the presence of H-αSynOs.

Since phenotypical changes in microglia involve changes in phagocytic function, and as we assumed that phagocytosis is the main clearance mechanism of extracellular p129-αSyn, we performed a functional assay in vitro to investigate whether H-αsynOs caused any alterations in microglial phagocytosis. Our data showed that the exposure of murine microglia (MMGT) to H-αsynOs for 24 and 48 h, but not for 6 h, reduced their phagocytic activity against fluorescent beads (*p* < 0.001 and *p* < 0.0001, respectively, by Tukey post hoc) (Figure 6A,C). This effect was dose-dependent (Figure 6B) and correlated with morphological changes, such as cell elongation and stronger vacuolization, evident 48 h after exposure to H-αsynOs (Figure 6D), suggesting that H-αsynOs impairs the phagocytic function of the microglia.

### 2.5. Nigrostriatal Degeneration

#### 2.5.1. Stereological Counting of TH+ Neurons

**Figure 7:**
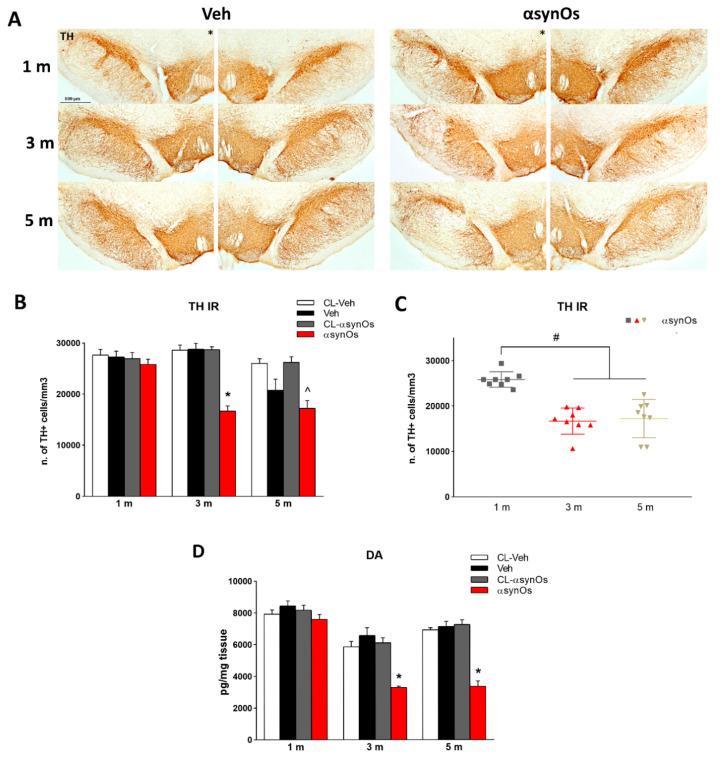
H-αSynOs induced a progressive nigrostriatal degeneration. Tyrosine hydroxylase (TH) immunostaining in the SNpc of vehicle and H-αsynO-infused rats (marked with *). (Magnification 5X) (**A**). Number of TH+ cells/mm^3^ measured by stereological counting, one, three, and five months after the H-αSynOs or vehicle infusion (**B**). Scatter plots shows the time-dependent progression of cell loss in the SNpc (**C**). Striatal dopamine content one, three, and five months after H-αSynOs or vehicle infusion (**D**). Values represent the mean ± SEM. * *p* < 0.0001 vs. all other groups in the same time point, ^ *p* < 0.0001 vs. CL-H-αSynOs, # *p* < 0.0001 vs. Veh and H-αSynOs one month post infusion (1 m) by Tukey post-hoc test (*n* = 8 per group).

Stereological analysis showed a gradual and persistent loss of dopamine neurons in the SNpc. One month post infusion of H-αsynOs the density of TH+ neurons in the infused SNpc was similar in all experimental groups, confirming that the infusion procedure did not cause any acute cell damage, as also shown by H&E staining performed three days after surgery (Figure 7A,B). Neuron density was decreased by 45% after three and five months (*p* < 0.0001 by Tukey post-hoc) (Figure 7A,B). As shown in Figure 7C, the density of TH+ cells was significantly lower at both three and five months post-infusion as compared with one month post infusion (*p* < 0.0001 by Tukey post-hoc).

#### 2.5.2. Striatal Dopamine

Figure 7D shows that H-αsynOs caused a non-significant reduction of dopamine in the ipsilateral striatum one month after the infusion (F_3,20_ = 0.26, *p* = 0.85), and a significant reduction after three months (F_3,38_ = 5.88, *p* < 0.005) and five months (F_3,20_ = 7,53, *p* < 0.005) (40% and 53%, respectively, when comparing the ipsilateral striata of H-αsynOs and vehicle-infused rats). The Tukey post-hoc analysis showed that dopamine tissue levels were significantly lower in the ipsilateral striatum of H-αsynOs-injected rats compared to the contralateral side (Figure 7A–C).

#### 2.5.3. Mitochondria

**Figure 8:**
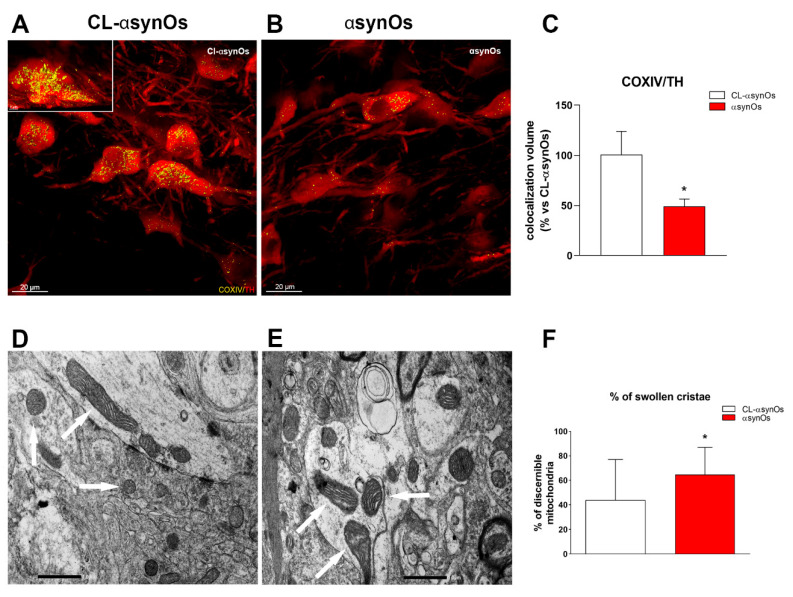
H-αSynOs induced early mitochondrial damage in the SNpc. Representative confocal images showing COX IV staining (yellow) in TH-positive cells (red) from the SNpc, contralateral (**A**) and ipsilateral (**B**) to H-αSynOs infusion, one month post infusion. Total volume occupied by COX IV in TH-positive cells (**C**); values are expressed as a percentage of contralateral (CL) side and represent the mean ± SEM. Representative images of mitochondria (10,000X, scale bar 1.0 μm) acquired with TEM from the SNpc, contralateral (**D**) and ipsilateral (**E**) to H-αSynOs infusion, one month post infusion. Arrows indicate mitochondria with normal cristae in A, and with swollen cristae in B; percentage of swollen cristae (**F**). * *p* < 0.01 vs. CL-H-αSynOs by t-test (*n* = 4 per group).

Since one month post infusion the presence of inflammatory microglia was not associated with any dopamine loss in the H-αsynOs-infused SNpc, we performed mitochondrial-mass measurement, specifically within TH-positive cells, and TEM morphometric analysis to investigate the potential loss or damage of mitochondria at this early time-point. COX IV-stained mitochondria were numerically reduced within TH-positive cells in the H-αsynOs-injected SNpc compared to the contralateral SNpc (Figure 8A–C); moreover, the TEM analysis revealed a significant increase in the number of swollen cristae, indicative of early ultrastructural damage (Figure 8D–F), (*p* < 0.01 by Student’s *t*-test).

### 2.6. Behavioral Assessment

#### 2.6.1. Beam Challenging Test

**Figure 9:**
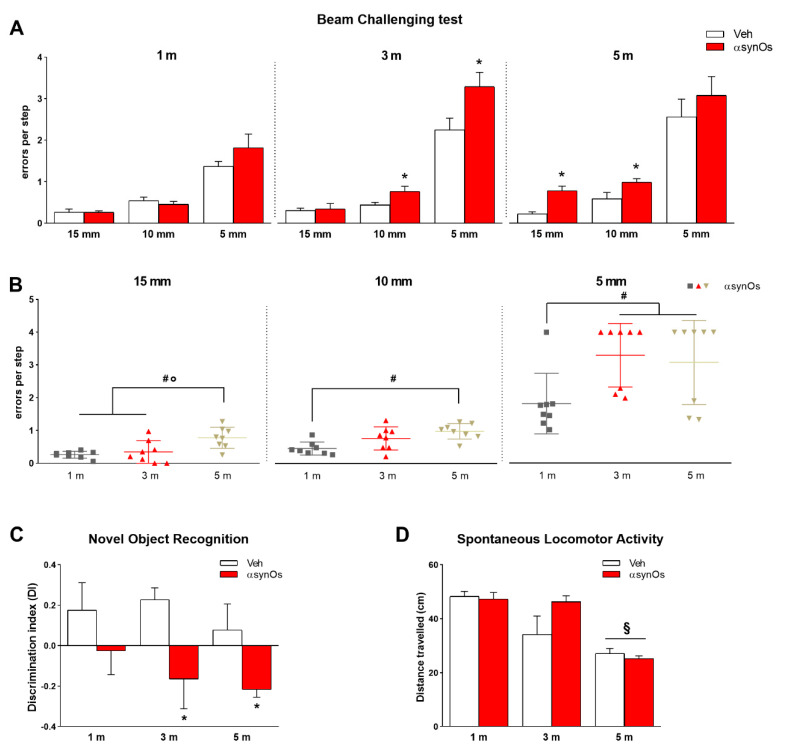
The H-αSynOs-infused rats developed motor impairments and cognitive deficits. Motor deficits displayed by Veh and H-αsynO-infused rats crossing beams of decreasing widths (15, 10, and 5 mm) at progressive time-points following infusion (one, three, and five months) (**A**). Scatter plots showing the time-dependent development of motor impairment for each beam (**B**). Motor impairment is expressed as the number of errors per step. Discrimination index measured by novel object recognition test (**C**), and spontaneous locomotor activity measured by the open field test, in Veh and H-αsynO-infused rats at progressive time-points (**D**). Values represent the mean ± SEM. * *p* < 0.05 vs. corresponding Veh; ^#^,° *p* < 0.05 vs. H-αSynOs one month and three months post infusion respectively (1 m and 3 m) by *t*-test; ^§^ *p* < 0.05 vs. the same experimental group one month post-infusion (*n* = 8 per group).

Motor performance was evaluated at progressive time points by means of the beam challenging test. H-αsynOs-infused rats displayed a consistent development of motor impairment that paralleled the nigrostriatal loss. Three-way ANOVA displayed the main effects of treatment (F_1,126_=13.0 *p* < 0.0005), width (F_2,126_ = 163.29 *p* < 0.000001), and time (F_2,126_ = 12.4 *p* < 0.00001) factors. Considering two-way ANOVA among each different time point, while at one month post-infusion of H-αsynOs the rats did not show any motor impairment in any tested beam (15,10,5 mm widths) (Figure 9A), they displayed a significant increase of errors per step when traversing the beams three (treatment: F_1,42_=8.43 *p* < 0.005; width: F_2,42_ = 91.5 *p* < 0.00001; treatment*width interaction F_2,42_ = 3.38 *p* < 0.05; 10 mm: t_14_ = 2.32 *p* < 0.05; 5 mm: t_14_ = 2.35 *p* < 0.05) and five (treatment: F_1,42_ = 5.01 *p* < 0.05; width: F_2,42_ = 44.4 *p* < 0.00001; 15mm t_14_ = 4.45 *p* < 0.005; 10 mm: t_14_ = 2.31 *p* < 0.05) months post-infusion (Figure 9A). Interestingly, when comparing the errors made by H-αsynOs infused rats in the same beam at progressive time points, we found a significant increase in the number of errors (one-way ANOVA for 15 mm: F_2,21_ = 7.89 *p* < 0.005; 10 mm: F_2,21_ = 7.56 *p* < 0.005; 5 mm beam width: F_2,21_ = 4.41 *p* < 0.05) (Figure 9B). Specifically, the Student *t*-test showed that the three and five monthinfused rats made a higher number of errors compared to the one month infused rats, in the 5 mm beam (t_14_ = 3.11 *p* < 0.005 and t_14_ = 2.24 *p* < 0.05 respectively). In addition, an increased number of errors was made by five month infused rats as compared to one and three month infused rats in the beam of 15 mm, suggesting a progression in the development of motor deficits (t_14_ = 4.32 *p* < 0.0005 and t_14_ = 4.32 *p* < 0.0005 respectively; Figure 9B).

#### 2.6.2. Novel Object Recognition Test

As shown in Figure 9C, H-αSynOs-infused rats showed a significant impairment of memory performance (discrimination index) as compared to the Vehicle-infused animals, which was detectable after one month, and became significant three- and five months, post infusion. Two-way ANOVA revealed the main effect of treatment (F_1,28_ = 11.50, *p* < 0.01). Multiple comparisons did not reveal significance between subjects. However, the subsequent Student’s *t*-test within each time point, showed significance at three and five months, when comparing vehicle vs. H-αSynOs-infused rats (t_14_ = 2.49, *p* < 0.05 and t_14_ = 2.19, *p* < 0.05 respectively).

#### 2.6.3. Open Field

The open field was used to measure the spontaneous locomotor activity of H-αSynO-infused and vehicle-infused rats at progressive time-points following the infusion (Figure 9D). Two-way ANOVA detected the main effect as time (F_2,28_ = 22.88, *p* < 0.0001). Bonferroni’s multiple comparisons test showed that in both vehicle and H-αSynO-infused rats the distance travelled decrease at five months after infusion, suggesting an age-dependent decrease on locomotor activity.

## 3. Discussion

We exploited the availability of well-characterized human H-αSynOs, which are kinetically trapped in a toxic conformation associated with a high homogeneity in size and structural properties [16]. We showed that the intranigral inoculation of these human H-αSynOs in rats induced a pathological scenario that recapitulated most neuropathological and symptomatic features of PD. The purity of these oligomers has previously enabled the characterization of the molecular mechanism by which they disrupt cellular membranes, which was tested in neuronal cells and *C. elegans* models of αSyn toxicity [16,24]. Here, we showed that the in vivo H-αSynOs infusion induced the early formation of p129-αSyn deposits in the ipsilateral SNpc that spread to the striatum, and was associated with mitochondrial loss and abnormalities in affected neurons, and with an early inflammatory response. P129-αSyn deposits preceded the progressive degeneration of dopaminergic cells and development of cognitive and motor impairment, which were consistently observed across the animals. A persistent neuroinflammatory response was observed up to the latest five month time-point investigated.

The presence of fibrillar aggregates of αSyn within LBs is a main hallmark of PD and synucleinopathies, however several pieces of evidence have suggested that the most toxic species of αSyn are small soluble aggregates in the form of oligomers, which form as intermediates during the process of amyloid fibril formation [4,9,10,11,33]. Accordingly, soluble oligomers have been described in the affected areas in the PD brain [34], as well as in the biological fluids of PD patients [35,36]. The close investigation of the structural features of αSyn has in recent years highlighted the high heterogenicity and transient nature of αSynOs. In vitro studies have largely elucidated the structure-related toxicity, showing that not only small size, but also specific features such as a highly lipophilic element, the exposure of hydrophobic residues on the αSynO surface, and a rigidly structured core, are key elements in eliciting the toxicity against neurons by facilitating non-physiological interactions with biological membranes [16,23,24]. Following this evidence, we reported the toxicity of oligomers with a small homogeneous size, of 12 to 14 nm in length and 9 to 10 nm in diameter, and carrying such toxicity-prone features [7]. The toxicity observed in our study was in line with previous findings showing that αSyn variants that showed impaired propensity to aggregate, and preferentially formed small oligomers, displayed the highest neurotoxicity in worms, flies, and mammalian neurons [10]; moreover, the lentivirus-mediated overexpression of αSyn variants that preferentially formed oligomers induced a more severe dopaminergic loss in the rat SNpc than the αSyn variants that induced rapid fibril formation, which were less toxic [4]. The intraventricular infusion of αSyn oligomers in mice induced a dopaminergic loss as early as 45 days after infusion, suggesting the elevated toxicity of oligomers [37]. Demonstrating the structure-related toxicity of αSyn, different αSyn strains inoculated into the olfactory bulb seeded and propagated the p129-αSyn pathology within the brain to different extents [27,38].

We showed here that the inoculation of purified human H-αSynOs induced the intraneuronal deposition of p129-αSyn in the infused SNpc as well as in the striatum, analyzed as the main dopaminergic projection area. The phosphorylation process accelerates the deposition of insoluble protein aggregates, and p129-αSyn is highly expressed within Lewy bodies [39]. Therefore, p129-αSyn is regarded as a marker of protein aggregation pathology [27,28]. In our study, deposits accumulated with different time-trends, remaining stable in the SNpc across time-points, while gradually increasing in number and size in the striatum after the H-αSynOs infusion. We used a rat/human antibody to detect p129-αsyn, suggesting that deposits were of endogenous origin. Therefore, exogenously inoculated H-αSynOs may have prompted endogenous αSyn in the infusion area to aggregate, followed by anterograde propagation along nigrostriatal axons [40,41,42]. Phosphorylation of Ser-129 has been associated with the αSyn oligomer accumulation and exacerbated the deposition of inclusions [43,44]. The development of p129-αSyn pathology in the affected dopaminergic neurons has been shown following the overexpression of αSyn, or the inoculation of αSyn fibrils [28,30,31,45,46,47]. Accordingly, the exposure of neurons from αSyn knockout mice to αSyn fibrils resulted in minimal p129-αSyn IR, confirming the need for recruiting endogenous αSyn in the aggregation process [48]. Moreover, previous studies have shown that distinct αSyn strains display differential spreading capacities, with oligomeric species holding the highest spreading potential; in agreement with a present study [12,45,46,49]. Interestingly, a recent study compared the in vivo p129-αSyn formation after inoculation of mouse αSyn fibrils or oligomers, suggesting that the former holds the greatest toxicity when injected into the mouse striatum [50]. Therefore, αSyn strains may display species-related toxicity, which adds a further order of complexity in the quest for structure-related toxicity. Supporting this concept, studies made on the virus-based model of αSyn overexpression reported that rodent αSyn did not induce any cell damage despite the presence of αSyn deposits within neurons, while the overexpression of the human form was neurotoxic [51]. Moreover, in vitro studies have demonstrated that the rodent αSyn aggregates into amyloid fibrils more rapidly than the human variant, which has the propensity to populate nonfibrillar soluble oligomers during the aggregation process [52].

Besides neuronal deposits, we found aggregates of p129-αSyn within microglial cells of the H-αSynO-infused SNpc, indicating that p129-αSyn was phagocyted by microglia. Interestingly, the colocalization volume of p129-αSyn within the microglia, and the size of the aggregates, were increased from one to three months post-oligomer infusion, but did not display any further increase at the later time point, suggesting that microglia phagocytosis of p129-αSyn was limited. Since a pathological interaction of αSyn with the phagocytosis machinery has been proposed in PD [53,54,55], we further investigated this issue via a functional in vitro assay, and showed that the exposure to H-αSynOs impaired the phagocytic efficiency of cultured microglia after prolonged, but not after short, incubation. The involvement of impaired phagocytic function in PD progression has been previously suggested, although not concordantly demonstrated [56]. Moreover, some studies suggested that phagocytosis was differently impaired by αSyn strains [57,58,59], while others reported a strain-independent induction of phagocytosis [57,60]. While our study confirmed that H-αSynOs alter the phagocytic capacity of microglia, further studies will be required to elucidate the underlying mechanism and whether oligomers are phagocytosed themselves or interact with pattern recognition receptors to alter microglia functions.

H-αSynOs triggered an intense neuroinflammatory response in the SNpc that preceded any frank cell loss and persisted in some aspects up to five months after the oligomer infusion, as shown by the early and increasing Iba-1 staining, early MHC-II staining, and changes in the cytokine profile. Specifically, we observed an early rise of the pro-inflammatory cytokine TNF-α and MHC-II, but not the anti-inflammatory cytokine IL-10, which instead displayed some reduction, indicating a disbalance in cytokine production, and suggesting that microglia acquired a pro-inflammatory profile after the interaction with H-αsynO, resembling PD neuropathology. Interestingly, MHC-II expression in the oligomer-infused SNpc was drastically decreased at three and five months, in line with previous reports [31], and despite the high TNF-α levels, suggesting that microglia lost the antigen presenting cell (APC) function following prolonged exposure to H-αSynOs, and yet maintaining a pro-inflammatory phenotype. The APC function is pivotal to the clearance efficiency of microglia, whose inhibition is fatal in neurodegenerative disorders [56,61]. In a previous study, we found a similar pattern of expression of the mannose receptor C (MRC) type I, a pattern recognition membrane receptor involved in phagocytosis, in MPTP intoxicated mice [62]. Altogether our data point to an αsynO-induced impairment in the phagocytic machinery, likely involving both defective phagocytosis and intracellular processing of the protein. Chronic microgliosis and altered cytokine profile are hallmarks of PD, as described by in vivo studies of the human PD brain [63,64,65]. A chronic inflammatory response that preceded and accompanied the dopaminergic degeneration has been described in toxin-based PD models [66,67,68,69]. However, models based on the AAV-mediated αSyn overexpression or the inoculation of PFFs reported only mild and transient microglial activation, while the cytokine levels were not investigated [16,28,70,71]. In vitro studies have shown that oligomeric species of αSyn display the greatest inflammatory potential when exposed to microglia, suggesting that toxic consequences of αSyn-microglia interaction is structure-related, in agreement with the present results [11,13,14,15].

H-αSynOs induced gradual damage and degeneration of dopaminergic cells within the SNpc, and a consistent decrease of dopamine content. Hence, dopamine neurons were not numerically decreased one month after the oligomer infusion, but displayed a reduced number and morphologically defective mitochondria, while they underwent cell death at later time points. Therefore, all together, the results revealed mitochondrial damage in dopamine neurons associated with an intense neuroinflammatory response in the SNpc that preceded neurodegeneration, suggesting that H-αSynOs may follow a double toxic pathway, directly interacting with mitochondria as previously shown in vitro [16,24,72], and inducing indirect, microglia-mediated neurotoxicity.

Reflecting the evolution of nigrostriatal degeneration, H-αSynOs-infused rats developed a significant motor impairment, that was unveiled by the beam challenging test. This is a highly sensitive test for the detection of sensorimotor deficits in the presence of partial nigrostriatal degeneration in αSyn-based models [73,74], and where the animal is exposed to increasingly challenging motor tasks by narrowing the beam width. We found that H-αSynO-infused rats incurred increasing motor faults along with the progression of neurodegeneration. Notably, both the dopaminergic loss and motor deficits were consistent across animals, suggesting an elevated degree of reproducibility. Interestingly, vehicle-infused rats displayed some motor deficits when facing the most challenging beam five months post-infusion, which may account for the lack of differences with oligomer-infused rats in this test. Moreover, the open field test failed to reveal any difference in spontaneous locomotion of H-αSynO-infused rats compared to the controls, which is in line with previous reports in αSyn models [45]. Instead, all rats showed a decrease in spontaneous locomotion five months post infusion of H-αSynO or vehicle. The rats were aged eight months at that time-point, suggesting that the age factor was adding to the oligomer toxicity. Interestingly, we found an impaired performance of oligomer-infused rats in the NOR test, indicating some degree of cognitive impairment, and suggesting that the present model deserves further characterization in relation to non-motor symptoms of PD and neuropathologically-related areas [75,76,77].

PD pathology following exogenous inoculation of PFFs has been intensively investigated in recent years [12,28,31,47,78,79]. The majority of studies addressing the toxicity of human αSyn in vivo have used PFF of heterogeneous length arising from the in vitro aggregation procedure. The heterogeneity of αSyn aggregates is a hampering factor for cross-study replication and protocol standardization, as these are associated with diverse structural and size distributions. Pre-formed fibrils of αSyn were associated with late nigrostriatal degeneration or no cell loss [28,30,49,79]. We showed here that the intracerebral inoculation of homogeneous and structurally characterized human αSyn oligomers provides a valid and reproducible model of the progressive neuropathology of PD in the rat, and resuming the main histopathological hallmarks and symptoms of this disease. Our results are particularly encouraging in view of the potential application for testing neuroprotective therapies against PD using this rat model. In particular, since the development of antibody-based therapies is a primary current interest in PD, the human H-αSynOs-based model presented here may provide a useful tool to test strain-specific clinically relevant antibodies.

## 4. Methods

### 4.1. Production of Recombinant H-αSyn

Recombinant H-αSyn was purified following overexpression in *Escherichia coli* using the plasmid pT7-7 [80]. After transforming in BL21 (DE3)-gold cells (Agilent Technologies, Santa Clara, USA), αSyn was obtained by growing the bacteria in at 37 °C under constant shaking at 250 rpm, and supplemented with 100 μg·mL^-1^ ampicillin to an OD600 of 0.6.

The expression was induced with 1 mM isopropyl β-D-1-thiogalactopyranoside (IPTG) at 37 °C for 4 h, and the cells were harvested by centrifugation at 6200× *g* (Beckman Coulter, Brea, CA, USA). The cell pellets were resuspended in lysis buffer (10 mM Tris-HCl pH 8, 1 mM EDTA, and EDTA-free complete protease inhibitor cocktail tablets obtained from Roche, Basel, Switzerland) and lysed by sonication. The cell lysate was centrifuged at 22,000× *g* for 30 min to remove cell debris. In order to precipitate the heat-sensitive proteins, the supernatant was then heated for 20 min at 70 °C and centrifuged at 22,000× *g*. Subsequently, streptomycin sulphate was added to the supernatant to a final concentration of 10 mg·mL^−1^, to stimulate DNA precipitation. The mixture was stirred for 15 min at 4 °C followed by centrifugation at 22,000× *g*. Then, ammonium sulphate was added to the supernatant to a concentration of 360 mg·mL^−1^, in order to precipitate the protein. The solution was stirred for 30 min at 4 °C and centrifuged again at 22,000× *g*. The resulting pellet was resuspended in 25 mM Tris-HCl, pH 7.7 and dialyzed against the same buffer in order to remove salts. The dialyzed solutions were then loaded onto an anion exchange column (26/10 Q sepharose high performance, GE Healthcare, Little Chalfont, UK) and eluted with a 0–1 M NaCl step gradient, and then further purified by loading onto a size exclusion column (Hiload 26/60 Superdex 75 preparation grade, GE Healthcare, Little Chalfont, UK). All the fractions containing the monomeric protein were pooled together and concentrated by using Vivaspin filter devices (Sartorius Stedim Biotech, Gottingen, Germany). The purity of the aliquots after each step was analyzed by SDS-PAGE, and the protein concentration was determined from the absorbance at 275 nm, using an extinction coefficient of 5600 M^−1^ cm^−1^.

### 4.2. Purification of H-αSynO

Toxic oligomeric samples were prepared from purified recombinant H-αSyn as previously described [16]. These oligomers have a relatively high homogeneity in size, as shown by analytical ultracentrifugation (AUC) and atomic force microscopy (AFM) [16]. The homogeneity of the oligomers also allowed for a structural characterization using solid-state NMR, including the definition of highly dynamical and rigid-core regions of the assemblies [16]. Briefly, 6 mg of lyophilized protein was resuspended in PBS buffer at a pH of 7.4, and at a concentration of 12 mg·mL^−1^. The solution was passed through a 0.22 μm cut off filter and subsequently incubated at 37 °C for 24 h in stationary mode, and without agitation in order to avoid acceleration of fibril formation. Residual fibrillar species were removed by ultracentrifugation for 1 h at 288,000× *g* using a TLA-120.2 Beckman rotor (Beckman Coulter, Brea, CA, USA). The excess of αS monomers in the sample was then removed by means of several filtration steps using 100 kDa cutoff membranes, which resulted in the enrichment of the oligomeric αS species. Samples of the toxic αSyn oligomers prepared in this manner have been found to be stable for many days, but in this study were used within two days of their production. In the case of the fluorescently labelled αSyn oligomers, labelled monomers carrying the AF488 dye (Invitrogen, Carlsbad, CA, USA) were obtained by using an N122C mutational variant, allowing the dye molecules to react with the thiol moiety of Cys122. The labeled protein was then purified from the excess of free dye by a P10 desalting column with a Sephadex G25 matrix (GE Healthcare, Waukesha, WI, USA) and concentrated using Amicon Ultra Centricons (Merck, Darmstadt, Germany). Fluorescent oligomers were generated by mixing 90% and 10% of unlabeled and labeled α-syn, respectively. The low ratio of labeled to unlabeled monomers, and the C-terminal position of Cys122 ensuring that both the core of the oligomers and the membrane interaction elements were unperturbed, guaranteed the absence of significant modifications to the properties of the oligomers.

### 4.3. Animals and Stereotaxic Surgery

Male Sprague Dawley rats (Envigo, Italy) were housed in groups of six in a climate-controlled animal room (21 ± 1°C; 60%) on a 12 h light/dark cycle (lights on at 7:00 a.m.) with standard chow and water ad libitum. All efforts were made to minimize animal discomfort and to reduce the number of animals used. All experimental procedures complied with the ARRIVE guidelines and were in accordance with the guidelines and protocols approved by the European Community (2010/63UE L 276 20/10/2010). Experimental protocols have been approved by the Italian Ministry of Health (Aut. 766-2020/PR).

In order to find the optimal volume and concentration of H-αsynOs, male Sprague Dawley rats weighing 275–300 g were deeply anesthetized with Fentanyl (3 mg/kg), and infused with 5, 10, and 20 μL of fluorescent oligomers at two different concentrations (1 mg/mL and 0.50 mg/mL) in the SNpc (anteroposterior: −5.4; mediolateral: −1.9; dorsoventral: −7.2) via a steel injector. Three days after surgery, the animals were transcardially perfused with PFA 4%, and the brains were collected and vibratome cut in 40 μm slices containing the SN. A set of slices across the SN were observed under a fluorescence microscope (Zeiss, 5X) to verify the infusion site, and to detect the extent of H-αsynOs diffusion within the SN or along the injector trace, and the deposition of large fluorescent aggregates that would prevent diffusion (Figure 2A). Moreover, H&E staining was performed to check for mechanical damage in the infused area (Figure 2B). The final infusion volume of 5 μL with 0.5 mg/mL H-αsynOs concentration was selected.

Therefore, for all subsequent experiments 87 rats were stereotaxically injected into the left SNpc with 5 μL of H-αsynOs (0.5 mg/mL, *n* = 45) or vehicle (Veh, *n* = 42) at the infusion rate of 1 μL/min (Figure 2B). The injector was left in place for an additional 5 min after infusion, and then slowly withdrawn.

### 4.4. Behavioral Tests

One, three, and five months post-surgery, behavioral tests were performed in order to detect motor deficits and cognitive impairment. Rats (*n* = 8 per group) were transferred to the test room 30 min before testing for acclimation, to avoid any alteration in behavioral parameters induced by the novel environment. Tests were carried out during the light time (9:00–14:00 h).

#### 4.4.1. Beam Challenging Test

Rats were tested by the beam challenging test, adapted from a previously developed protocol [73], in order to assess motor deficits. The testing apparatus consisted of 2 m-long wooden beams placed between a starting platform, elevated 40 cm from the floor, and the home cage, with a slope of 15°. Three different beam widths were used: 15, 10, and 5 mm. Rats were trained for three days to walk along the beam, and on the test day they were videotaped. Briefly, the rat was placed at the lower end of the beam and the number of stepping errors was counted while traversing the beam to reach the home cage. The same procedure was repeated for the three different widths. If the animal was not able to complete the task in 120 sec or if it fell off the beam, the maximum error score was assigned.

#### 4.4.2. Spontaneous Locomotor Activity

The open field test was performed to measure spontaneous locomotor activity. Rats were recorded by a videocamera to track the animal position and movements, and data were analyzed by Anymaze behavioral tracking software. The rats were individually placed in the center of a squared arena (100 × 100 cm), and spontaneous motor activity was monitored for 10 min (test period).

The total distance travelled was analyzed and expressed in cm, which represented the horizontal distance travelled by an animal.

#### 4.4.3. Novel Object Recognition Test (NOR)

A NOR test was carried out according to a previously published protocol [75]. Briefly, after the habituation session (10 min), each animal was placed in the box (60 × 60 cm) and allowed to explore two identical objects for 10 min (familiarization phase, T1). The second trial (choice phase, T2) was performed after a 1 h interval, when the animal was exposed to the same condition as in T1, except that one object was replaced with a new one (novel object). All objects were thoroughly cleaned across trials to avoid olfactory cues. The latency of the first approach (time taken to approach any of the two objects), frequency of approaches (number), time (sec) spent in the exploration of familiar and novel object were recorded, and the discrimination index ((Tn-Tf)/(Tn+Tf) was calculated.

### 4.5. Immunohistochemistry

After the behavioral tests, the rats were anesthetized and transcardially perfused with 4% paraformaldehyde. The brains were post-fixed and 40 µm thick coronal midbrain and striatum sections were vibratome-cut.

For stereological counting (*n* = 8 per group), midbrain sections were pre-incubated with a blocking solution with normal serum, and then immunoreacted with polyclonal rabbit anti-TH (1:1000, Millipore, Burlington, MA, USA) primary antibody. TH-positive cells were visualized using the classic avidin-peroxidase complex (ABC, Vector, UK) protocol, using 3,30-diaminobenzidine (Sigma-Aldrich, St. Louis, MO, USA) as a chromogen.

For immunofluorescence (*n* = 4–5 per group), 3 sections per each animal, from the midbrain or the striatum, were pre-incubated with a blocking solution with normal serum/BSA, and then immunoreacted with primary antibodies for single or double immunolabelling: goat polyclonal anti Iba-1 (1:1000; Novus Biologicals, Littleton, Colorado, US); rabbit polyclonal anti-TNF-α (1:500, Novus Biologicals, Littleton, CO, USA); rabbit polyclonal anti IL-10 (1:200, Abbiotec, Escondido, CA, USA); rabbit monoclonal anti p129-αSyn (1:800, Abcam, Cambridge, UK); mouse monoclonal anti-TH (1:400, Millipore, Burlington, MA, USA); rabbit polyclonal anti COX IV (1:500, Invitrogen); mouse monoclonal anti MHC-II (1:150, Abcam, Cambridge, UK). Control slices were incubated without primary antibodies, and all slices were thereafter incubated with the appropriate fluorochrome-conjugated secondary antibodies. For fluorescence visualization of Iba-1 a two-step indirect labelling protocol was used, while a three-step detection was performed to increase the signal of TNF-α, IL-10, and p129-αSyn, as previously described [81].

For thioflavin S staining, midbrain sections were incubated for 8 min with 0.05% Thioflavin S (Sigma-Aldrich, St. Louis, MO, USA) dissolved in 50% EtOH, and then washed three times in EtOH 80% in order to eliminate unspecific dye residues, as described [82]. Sections were then immunoreacted with monoclonal mouse anti-TH (1:400, Millipore, Burlington, MA, USA); goat polyclonal anti Iba-1 (1:1000, Novus Biologicals, Littleton, CO, USA), as described above for single immunolabeling. Images were acquired using a laser scanning confocal microscope (Zeiss, Oberkochen, Germany) with a 63× magnification.

### 4.6. Stereological Counting of TH Immunoreactivity

All immunohistochemical reactions were analyzed by an operator blind to the experimental groups, and different from the experimenter that performed the behavioral tests and histology. TH-immunoreactive neurons were counted bilaterally in the SNc, as previously described [83]. A dedicated software was used (Stereologer, System Planning and Analysis, Inc., Alexandria, VA, USA), linked to a motorized stage on a BX-60 Olympus light microscope (Olympus, Segrate, Italy). The total number of TH-stained cells was estimated by means of optical fractionator method, which combines the optical dissector with the fractionator sampling scheme, giving a direct estimation of the number of 3-D objects unbiased by shape, size, and orientation [84]. A systematic random sampling of cells within the area of interest was achieved by “Stereologer” software. Equidistant counting frames (frame area = 50 μm^2^) were obtained. Sampling fraction was delimited at low power and cells were sampled with a ×40 oil immersion objective through a defined depth with a 2 μm guard zone. The coefficient of error (CE) for each estimation and animal ranged from 0.05 to 0.1.

### 4.7. Microscopy Analysis

Qualitative and quantitative analysis for markers of neuroinflammation and p129-αSyn was performed on immunofluorescence-stained sections by an operator blind to the experimental groups, using a spinning disk confocal microscope (Crisel Instruments, Rome, Italy) with a 63X oil objective. The analysis for p129-αSyn colocalized with Iba-1 and TH was performed using a laser scanning confocal microscope Zeiss with a 63X oil objective. Each frame was acquired eight times and then averaged to obtain noise-free images. Surface rendering, maximum intensity, colocalization, and simulated fluorescence process algorithms were used (ImageJ and Imaris 7.3). 3D reconstruction of neuron body and fibers, as well as microglia, containing p129-αSyn is shown in supp. Figure 1.

The volumes occupied by Iba-1, MHC-II, TNF-α/Iba-1, IL-10/Iba-1, p129-αSyn/Iba-1, p129-αSyn/TH, COX IV/TH colocalization in SNpc, and p129-αSyn/TH in the dorsal striatum, were determined. For colocalization analysis, a colocalization channel was automatically generated by Imaris 7.3. A stack was obtained from each dataset (20–40 images). In the resulting stacks, ten regions of interest for the SNpc (x = 700 μm; y = 700 μm; z = 40 μm) and five regions of interest for the dorsal striatum (x = 1024 μm; y = 1024 μm; z = 40 μm) in each acquired section and for each animal were randomly chosen, and volume of the elements calculated. Values were expressed as a volume. The sides contralateral to the stereotaxic infusion were analyzed as inner controls across studies.

### 4.8. Striatal Dopamine Assessment

Striatal tissue (*n* = 6 per group) was sonicated in 0.25 mL of 0.2 M perchloric acid and then centrifuged at 9391× *g* for 15 min at 4 °C. The supernatant was transferred and filtered in a Spin-X centrifuge tube filters (0.45 mm). The filtrate was diluted 1:10. Twenty microliters were injected into an HPLC apparatus, equipped with a rever se-phase column (LC -18 DB, 15 cm, 5 lm particle size; Supelco, Milano, Italy) and a coulometric detector (ESA Coulochem IIm, Bedford, MA, USA) to quantitate DA. Electrodes were set at +150 mV (oxidation) and 250 mV (reduction). The mobile phase (nM composition was: NaH2PO4, 100; NA2EDTA, 0.1; n-octyl sodium sulphate, 0.5; 7.5% methanol; pH 5.5) was pumped (Jasco Europe, Cremella, Italy) at 1 mL/min flow rate. The assay sensitivity for DA and DOPAC was 10 fmol/sample.

### 4.9. Electron Microscopy and Mitochondria Analysis

One month post infusion, H-αsynOs-infused rats (*n* = 4) were transcardially perfused with a mix of 1% paraformaldehyde and 1.25% glutaraldehyde in 0.15 M cacodylate buffer. After fixation and rinsing in the same buffer, brains were post-fixed with 1% osmium tetroxide in distilled water for 2 h, and then stained overnight with 0.5% uranyl acetate at 4 °C. The brains were then dehydrated in a graded acetone series and embedded in EPON resin. To identify the SNpc, semi-thin coronal sections of the SN were cut with a Reichert Supernova ultramicrotome and stained with toluidine blue. Ultrathin sections (90 nm, at least 5 sections for each rat) were observed under a JEOL JEM 1400 Plus electron microscope, equipped with a CCD camera, at an acceleration voltage of 80 kV.

The total number of mitochondria, the percentage of swollen cristae mitochondria (expressed as the percentage of mitochondria with swollen cristae on the total mitochondria with discernible cristae in the sampled area) was evaluated in the unitary area (25 µm^2^) within the SNpc infused with H-αsynOs and the contralateral SNpc as a control. A total of 40-50 unitary area fields were considered in each hemisphere. More than 6700 mitochondria were randomly sampled on 505 non-overlapping micrographs at a final magnification of 10,000×. Swollen cristae were recognized when the individual cristae size (i.e., the distance between two contiguous membranes of one cristae) doubled the average cristae size.

### 4.10. Phagocytosis Assay

The murine microglial cell line MMGT12 was used. Cells were cultured in DF culture medium comprising DMEM/F12 (1:1, vol/vol), supplemented with 10% fetal bovine serum (FBS) without antibiotics, grown in humidified atmosphere of 5% CO_2_ at 37 °C, and harvested and seeded twice a week. MMGT12 cells were seeded on 24-well plates at the density 5 x 10^4^ cells per well, and α-syn oligomers (0.6 μm) were added 2, 26, and 44 h after seeding. Control cells were supplemented with equivalent volume of vehicle at the same times. Then, 48 h after the first treatment all the cells were tested for phagocytotic capacity as indicated below. Alternatively, the cells were incubated with different concentrations of α-synOs the day after seeding for 24 h as previously described [62]. Briefly, cell monolayers were washed in PBS, trypsinized in 0.05% trypsin for 2 min and re-plated in the same wells, using the same medium, for one hour. Fluoresbrite carboxy YG 6.0 micron beads (Polysciences Inc., Warrington, PA, US, cat#18141) were resuspended in PBS with 5.5 mM glucose, 1.5 mM MgCl_2_, and 1 mM CaCl_2_, and pre-opsonized by addition of 50% FBS and incubation for 30 min in 5% CO2 at 37 °C. 12 × 10^6^ pre-opsonized microspheres were resuspended in 12 mL of DF culture medium without FBS, distributed on cells (around 5 beads/cell), and incubated for 2.5-3 h. Cells were washed with PBS, trypsinized for 4 min and collected with DF medium in conical tubes, washed with PBS, and suspended in 0.3 mL PBS containing 1% FBS and 0.05% EDTA. Samples were acquired in the green channel (502 nm) by FACS Aria (BD Biosciences, Erenbodgem, Belgium) within 20 min. The whole experiment was repeated three times.

### 4.11. Statistical Analysis

Statistical analysis was carried out by Statistica 8 (Stat Soft Inc., Tulsa, OK, USA). Behavioral data were presented as mean ± SEM and were analyzed by three-, two- or one-way analysis of variance (ANOVA), with groups, months, and width as factors. Post-hoc comparisons were made using Tukey, Bonferroni multiple comparison test, or t test, where appropriate. Results were analyzed using GraphPad Prism^®^ 6 for Windows (GraphPad software). Histological, HPLC, and in vitro data were statistically compared by one-way ANOVA followed by Tukey post-hoc test, or by Student’s *t*-test for electron microscopy, as data did not follow a normal distribution. The level of significance was set at *p* < 0.05.

## Supplementary Materials

Supplementary materials can be found at https://www.mdpi.com/1422-0067/21/22/8535/s1.
