# Supplementary material for: Modeling Parkinson’s Disease Neuropathology and Symptoms by Intranigral Inoculation of Preformed Human α-Synuclein Oligomers
Source: Int J Mol Sci. 2020 Nov 12;21(22):8535. doi: 10.3390/ijms21228535 (PMC7696693; doi:10.3390/ijms21228535)
Supplement: Supplementary file 1 [file ijms-21-08535-s001.pdf]

Supplementary Figures

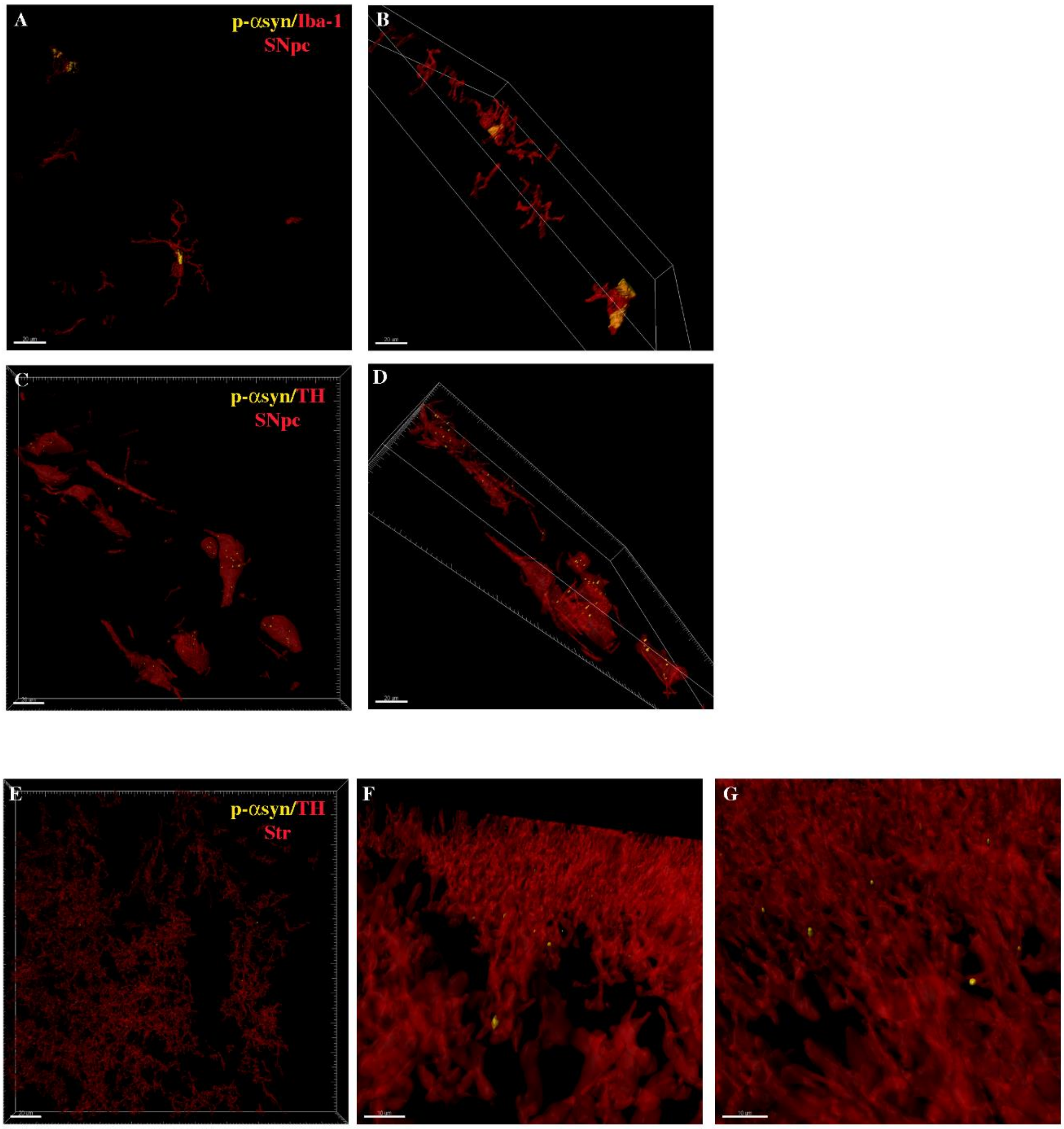

Figure S1. 3D reconstruction of colocalization of p129-αSyn (yellow) with Iba-1 (red, A-B) or TH cell bodies (red, C-D) and fibers (E-G), revealed by surface rendering algorithm in SNpc. To evaluate the p129-αSyn primary and secondary antibodies penetration into the cell, the image stack was rotated of about 45° (B, D) or 9° (F, G) in the Y-axis. All scales are expressed in µm.

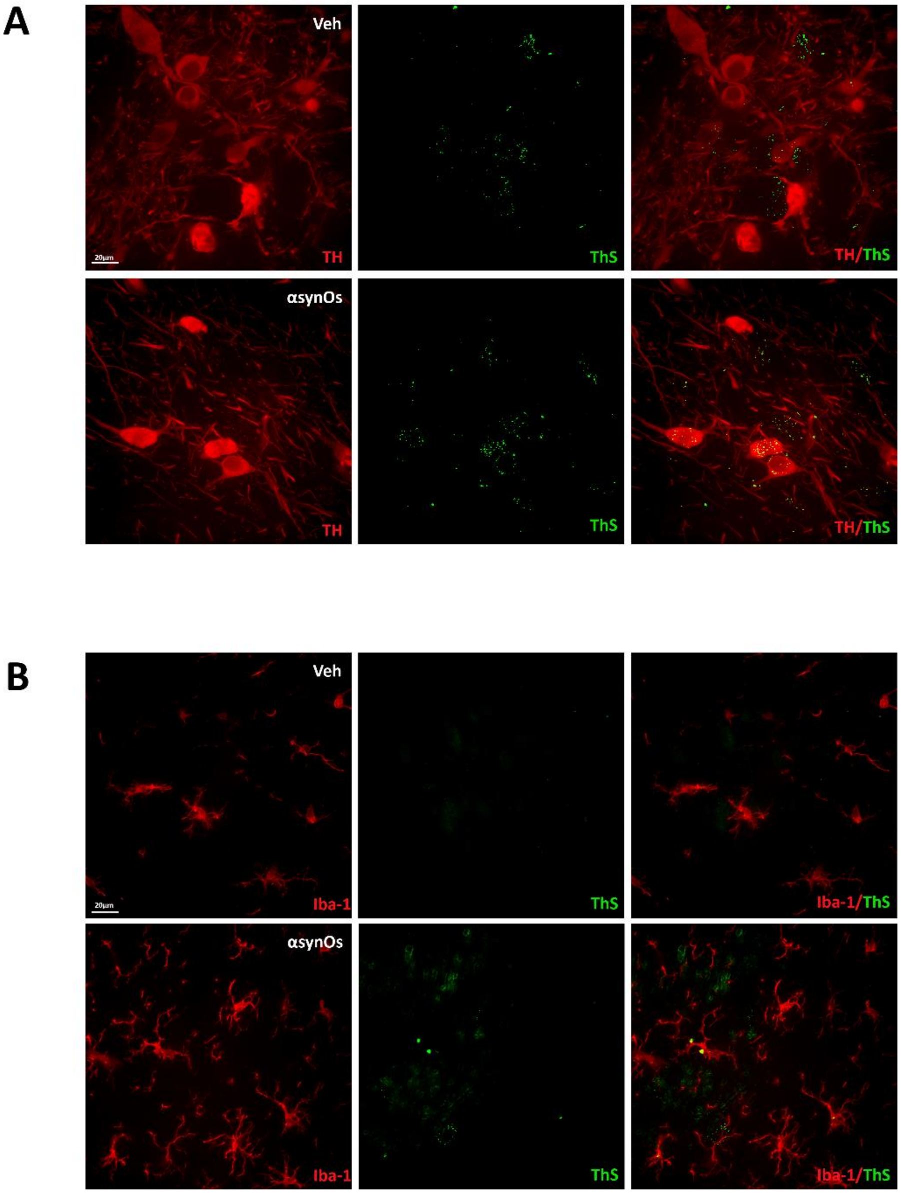

Figure S2. Thioflavin-S signal in TH-positive and Iba-1 positive cells. Representative confocal images from the Substantia Nigra pars compacta (SNpc) of vehicle or H-αSynOs-infused rats. Thioflavin-S aggregates (green) are shown in TH^+ neurons (red in A panels) and Iba-1 positive cells (red in B panels). Scale bar: 20µm.

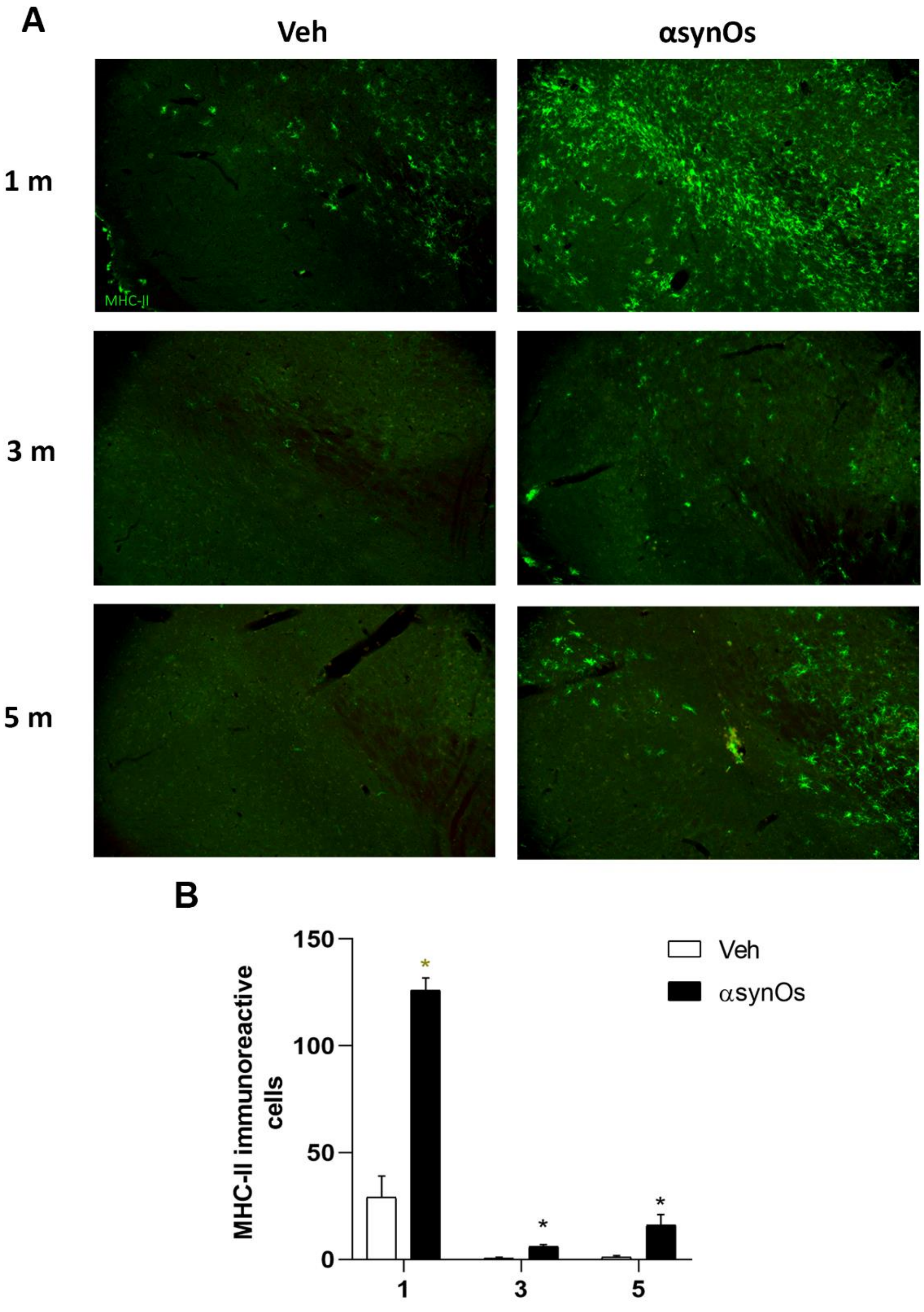

Figure S3. Representative images of MHC-II immunofluorescence (A) and quantification of MHC-II-immunoreactive cells (B) in the SNpc from vehicle or H-αSynO-infused rats (A). Values represent the mean ± SEM. *p<0.01 vs corresponding vehicle one, three or five months post infusion, by t-test (n=4/5 per group).
